# Performance comparison of BD Phoenix CPO detect panel with Cepheid Xpert Carba-R assay for the detection of carbapenemase-producing *Klebsiella pneumoniae* isolates

**DOI:** 10.1186/s12866-024-03311-7

**Published:** 2024-05-17

**Authors:** Nazmiye Ülkü Tüzemen, Uğur Önal, Osman Merdan, Bekir Akca, Beyza Ener, Halis Akalın, Cüneyt Özakın

**Affiliations:** 1 Faculty of Medicine, Department of Medical Microbiology, Bursa Uludag University, Görükle, Bursa, 16059 Turkey; 2 Faculty of Medicine, Department of Infectious Diseases and Clinical Microbiology, Bursa Uludag University, Bursa, Turkey; 3 Technical University of Munich, Institute of Virology, Munich, Germany

**Keywords:** *Klebsiella pneumoniae*, Carbapenem-resistant, BD Phoenix CPO, Xpert Carba-R assay

## Abstract

**Background:**

We aimed to compare the performance of carbapenemase classification in carbapenem-resistant *Klebsiella pneumoniae* (CRKP) obtained using the BD Phoenix CPO Detect panel (CPO panel) and Cepheid Xpert Carba-R assays. We analyzed 55 CRKP strains from clinical specimens collected between November 2020 and November 2022. The CPO panel was used to detect both antibiotic susceptibility and phenotypic carbapenemase classes, while Xpert Carba-R was employed to identify KPC, NDM, VIM, OXA-48, and IMP genes. Due to the limited availability of molecular kits, we arbitrarily selected 55 isolates, identified as carbapenemase-producing according to the CPO panel and with meropenem minimum inhibitory concentration values > 8 mg/L.

**Results:**

According to the Xpert Carba-R assay, 16 of the 55 isolates (29.1%) were categorised as Ambler Class A (11 of which matched CPO panel Class A identification); three isolates (5.5%) were identified as Class B and 27 isolates (49.1%) as Class D (in both cases consistent with CPO panel B and D classifications). A further eight isolates (14.5%) exhibited multiple carbapenemase enzymes and were designated as dual-carbapenemase producers, while one isolate (1.8%) was identified as a non-carbapenemase-producer. The CPO panel demonstrated positive and negative percent agreements of 100% and 85.7% for Ambler Class A, 100% and 100% for Class B, and 96.4% and 100% for Class D carbapenemase detection, respectively.

**Conclusion:**

While the CPO panel’s phenotypic performance was satisfactory in detecting Class B and D carbapenemases, additional confirmatory testing may be necessary for Class A carbapenemases as part of routine laboratory procedures.

## Introduction

Antimicrobial resistance in Gram-negative bacteria, particularly *Enterobacterales*, poses an increasingly severe threat to global public health. The list of pathogens published by The World Health Organization (WHO) includes those designated by the acronym ESKAPE (*Enterococcus faecium, Staphylococcus aureus, Klebsiella pneumoniae, Acinetobacter baumannii, Pseudomonas aeruginosa*, and *Enterobacter* spp.); often known as ‘superbugs’, they can acquire high resistance levels [1]. Carbapenemase-producing Enterobacterales (CPE) are a primary public health concern since carbapenems are considered one of the options for treating multidrug-resistant (MDR) Gram-negative bacterial infections [2, 3], and so rapid and accurate carbapenemase identification is essential for epidemiological and infection control purposes [4]. Currently, *K. pneumoniae* carbapenemase (KPCs), an Ambler Class A genes, is the most common transmissible gene circulating in Enterobacteriaceae worldwide [5]. Moreover, the Centers for Disease Control and Prevention (CDC) has reported an alarming increase in the rate of CPE commencing during hospitalization, a rise of more than 35% from 2019 to 2020 in the United States (US) [6].

There are three main mechanisms causing carbapenem resistance in Enterobacterales. The first one is enzyme production, such as extended-spectrum beta-lactamases (ESBLs), metallo-beta-lactamases (MBLs), and other categories of carbapenemases. The second is efflux pumps, and the third is porin mutations. Carbapenem-resistant Enterobacterales (CRE) are further divided into CPEs and non-CPEs. CPEs can produce a large variety of carbapenemases, which can be divided into three groups according to the Ambler classification: Class A (serine-β-lactamases), Class B (MBLs), and Class D (oxacillinases; OXAs) ß-lactamases. The clinical relevance of a fourth grouping, Ambler Class C (AmpC β-lactamase) enzymes, remains to be determined [7]. MDR *Enterobacteriaceae* is thought to have initially emerged in the 1980s, shortly after the use of cephalosporins and other broad-spectrum ß-lactam antibiotics became widespread. According to epidemiological data, the prevalence of various CPEs varies with geographical region [8].

Phenotypic and molecular-based techniques are the two main methods currently used to detect carbapenemases. The modified Hodge test (mHT), Triton Hodge test, Carba NP test, Blue-Carba test, and modified carbapenem inactivation method (mCIM) are some phenotypic carbapenemase detection assays. The matrix-assisted laser desorption ionization–time of flight mass spectrometry (MALDI–TOF MS) can be used to identify for CPE. Molecular techniques are currently considered to be the gold standard for the identification of carbapenemase genes. PCR is the most common method for traditional molecular genotyping [9].

BD launched the BD Phoenix CPO Detect panels (CPO panel) in 2017. These panels are used for susceptibility testing, phenotypic carbapenemase detection, and classification according to Ambler classes. For detecting and classifying carbapenemases, panels utilize nine wells containing meropenem, doripenem, temocillin, and cloxacillin, either alone or in combination with various chelators and beta-lactamase inhibitors. The panels are able to detect phenotypic carbapenem resistance [10]. In diagnostic laboratories, the ability to determine the presence and classification of carbapenemases without requiring any additional method, while also obtaining the results of antibiotic susceptibility tests, is highly advantageous.

Despite the limited number of studies conducted in our country compared to more developed nations, it has become evident that we also have serious carbapenem resistance. In light of this issue, we aimed to compare the performances of the CPO panel and the Cepheid Xpert Carba-R assay in detecting carbapenemase classes of carbapenem-resistant *Klebsiella pneumoniae* (CRKP).

## Materials and methods

### Study design and bacterial strains

This single-center study was conducted at a 900-bed tertiary care university hospital, the largest one in the Southern Marmara region, in line with the principles of the Declaration of Helsinki.

Samples isolated from various clinical specimens sent to the Medical Microbiology Laboratory of our hospital between November 2020 and November 2022 were identified by MALDITOF MS (Bruker Daltonik, Bremen, Germany) (IVD v 12.0 database); those with a score of two or higher were scanned. These isolates were subcultured onto 5% sheep blood agar and eosin methylene blue agar (BD Diagnostic Systems) and incubated at 37 °C overnight. A second subculture was performed before testing.

Because of the limited availability of molecular kits, we arbitrarily selected 55 isolates, one isolate per patient, whose meropenem minimum inhibitory concentration (MIC) value > 8 mg/L (resistant according to the European Committee on Antimicrobial Susceptibility Testing [EUCAST]) were included in the study. We selected meropenem-resistant isolates because we could not compare them with the CPO panel if the gene could not be detected by the molecular kit. Only adults who were inpatients at our hospital were included, with one isolate per patient taken for further analysis.

A total of 284 *K. pneumoniae* isolates were collected for this study. They were all detected using the CPO (NMIC/ID-505) panel. 179 of the isolates had a meropenem MIC value of > 8 mg/L, and 163 were from adults. 127 were identified as phenotypic carbapenemase producers by the CPO panel. When repeat samples were excluded, 94 samples remained. Of these, 55 samples were selected arbitrarily, prioritizing blood, respiratory tract, and sterile body fluid samples.

### BD Phoenix CPO panel

Antibiotic susceptibility testing was performed using the BD Phoenix™ M50 System according to EUCAST recommendations [11]. Our laboratory employs the BD Phoenix Gram-negative (NMIC/ID-433) panel for Gram-negative isolates except for urine isolates. For patients in the Hematology, Oncology, and Intensive Care Units (ICU), we utilize the CPO (NMIC/ID-505) panel. The isolates used for the CPO panel as part of antimicrobial susceptibility testing (AST) panels were included in the study. This CPO panel is designed to detect carbapenemase activity and classify carbapenemase producers according to Ambler classification. It uses nine test wells on the Phoenix panel, each containing a β-lactam antibiotic alone or combined with various β-lactamase inhibitors, for algorithm-based detection and classification of carbapenemase-producing organisms. The EpiCenter software (Becton Dickinson) measures values and interprets the results. For the CPO panel, EpiCenter delivers two results according to its algorithm by interpreting the test wells: the carbapenemase activity and, when is positive, the carbapenemase type based on Ambler Classes A, B, and D. It is crucial to understand that the CPO panel test is not capable of identifying both types of carbapenemase produced by some bacterial isolates. This test only provides results for one class of carbapenemase, and cannot detect dual carbapenemase class results. Furthermore, there are cases where the carbapenemase producers may not be classified at all [12, 13]. Our study used the reference strain *K. pneumoniae* ATCC 700,603 to ensure quality control.

### Xpert Carba-R assay

We used Xpert Carba-R assay v. 2 (Cepheid, Sunnyvale, CA, USA), a multiplex real-time PCR assay for molecular testing. It is a qualitative, real-time PCR test for detecting and differentiating KPC, NDM, VIM, OXA-48, and IMP in 48 min from pure bacterial colonies. This automated system integrates sample preparation, nucleic acid extraction, amplification, and target sequence detection [14]. The assay was conducted according to the manufacturer’s instructions.

### Statistical analysis

We calculated the obtained data’s absolute and relative frequencies for statistical analysis. Additionally, we determined positive percent agreement (PPA), negative percent agreement (NPA), and kappa values by using the IBM SPSS version 28.0 (Armonk, NY: IBM Corp.) A *p*-value below 0.05 was considered to be statistically significant. PPA is calculated using identical formulae to sensitivity, and NPA is calculated identically to specificity [15]. Kappa results for agreement can be interpreted as follows: values ≤ 0 indicate no agreement, 0.01–0.20 from none to slight, 0.21–0.40 as fair, 0.41– 0.60 as moderate, 0.61–0.80 as substantial, and 0.81–1.00 as almost perfect agreement [16].

## Results

In our study, a total of 55 CRKP strains, each belonging to a different patient, were grown from clinical specimens collected between November 2020 and November 2022. The distribution of the samples was as follows: blood (25 isolates, 45.5%), deep tracheal aspirate (18 isolates, 32.7%), wound pus (6 isolates, 10.9%), sputum (3 isolates, 5.5%), urine (2 isolates, 3.6%), and cerebrospinal fluid (1 isolate, 1.8%).

The CPO (NMIC/ID-505) panel detects and classifies phenotypic carbapenemases. We intentionally selected meropenem-resistant, carbapenem-producing isolates because if the gene could not be found with the molecular kit, we would not be able to make comparisons using the CPO panel; hence, all 55 strains were carbapenemase producers.

All dual carbapenemases detected by Xpert Carba-R assay were also identified as producing carbapenemases in the CPO panel. However, the CPO panel only provides results for one class of carbapenemase, not double carbapenemase class results. To ensure a more accurate performance analysis of the CPO panel, we excluded the dual-resulting samples identified in the Xpert Carba-R assay, except for blaNDM and blaVIM dual-positivity cases since both belong to the same Ambler Class. Consequently, we were able to analyze the performance of the CPO panel using a total of 46 samples (Table 1). In comparisons with the CPO panel, the Xpert Carba-R assay showed a kappa value of 0.804 (*p* < 0.001) for the detection of total carbapenemase classes. Meanwhile, the kappa value for class A in the CPO panel showed substantial agreement, while the kappa value for total and classes B and D demonstrated perfect agreement.

**Table 1:** BD Phoenix CPO panel performance, with indeterminate results excluded, for Ambler classification, compared to Xpert Carba-R assay results

Ambler Class (Xpert Carba-*R*)	No. Tested	No. of BD Phoenix CPO panel results				Results							
		TP	FP	FN	TN		PPA (%, 95%CI)		NPA (%, 95%CI)	Kappa value		Significance	
A	16	11	0	5	30			100 (71.5–100)	85.7 (74.3–92.5)	0.742		< 0.001	
B	3	3	0	0	43			100 (29.2–100)	100 (91.7–100)	1		< 0.001	
D	27	27	1	0	18			96.4 (80.3–99.4)	100 (81.4–100)	0.955		< 0.001	

TP: True Positive, FN: False Negative, TN: True Negative, FP: False Positive, PPA: Positive Percent Agreement, NPA: Negative Percent Agreement, CI: Confidence Interval

Among the initial 94 isolates, the CPO panel identified 42 (44.7%) as Class A, 3 (3.2%) as Class B, and 49 (52.1%) as Class D carbapenemases. However, due to budget constraints, the original 94 isolates that met the study criteria were narrowed down to 55, prioritizing blood, respiratory tract samples, and sterile body fluid samples.

Table 2 presents the detailed classification results of the narrowed down 55 isolates from both the CPO panel and the Xpert Carba-R assay. Of the 55 isolates, the CPO panel classified 14 (25.5%) as Ambler Class A carbapenemases (CARBA), 3 (5.5%) as Class B carbapenemases (CARBB), 30 (55.5%) as Class D carbapenemases (CARBD), and 8 (14.5%) as carbapenemase producers with no specific classification (CARB). Meanwhile, the Xpert Carba-R assay labeled 16 isolates (29.1%) as CARBA (11 of which were previously identified as Class A in the CPO panel), three isolates (5.5%) as CARBB (all initially recognized as Class B by the CPO panel), and 27 isolates (49.1%) as CARBD (also identified as Class D in the CPO panel). Moreover, eight isolates (14.5%) demonstrated the presence of multiple carbapenemase enzymes and were classified as dual-carbapenemase (D-CARB), while one isolate (1.8%) was categorized as a non-carbapenemase producer (non-CARB) by the Xpert Carba-R assay (also previously identified as a non-carbapenemase-producer in the CPO panel). Furthermore, 6 isolates (10.9%) showed the presence of both CARBA and CARBD, 1 isolate (1.8%) exhibited CARBB and CARBD, and 1 isolate (1.8%) displayed the presence of CARBA, CARBB, and CARBD simultaneously (Table 2).

**Table 2:** Comparison of the results of carbapenemase classification between BD Phoenix CPO panel and Xpert Carba-R assay

Classification Carbapenemase by Xpert Carba-*R* assay		Carbapenemase enzyme (Xpert Carba-*R* assay)	No. Tested *n* (%)	Classification Carbapenemase by BD Phoenix CPO panel			
				CARBA *n* (%)	CARBB *n* (%)	CARBD *n* (%)	CARB *n* (%)
CARBA		KPC	16 (29.1)	11 (20)	-	1 (1.8)	4 (7.3)
CARBB		NDM	2 (3.7)	-	2 (3.7)	-	-
		VIM + NDM	1 (1.8)	-	1 (1.8)	-	-
CARBD		OXA-48	27 (49.1)	-	-	27 (49.1)	-
D-CARB	CARBA + CARBD	KPC + OXA 48	6 (10.9)	3 (5.5)	-	1 (1.8)	2 (3.6)
	CARBB + CARBD	NDM + OXA-48	1 (1.8)	-	-	1 (1.8)	-
	CARBA + CARBB + CARBD	NDM + KPC + OXA-48	1 (1.8)	-	-	-	1 (1.8)
non-CARB		-	1 (1.8)	-	-	-	1 (1.8)

CARBA: Ambler Class A, CARBB: Ambler Class B, CARBD: Ambler Class D, CARB: carbapenemase-producer without further details, D-CARB: Dual carbapenemase-producer, non-CARB: non-carbapenemase-producer

## Discussion

Antibiotic resistance has emerged as a significant global health concern over the last two decades. Among the Enterobacteriaceae family, which includes ESBL-producing organisms, carbapenem antibiotics have provided the most effective treatment option [17]. However, the rise in carbapenem resistance, particularly in *K. pneumoniae*, has become a significant challenge over the last several years, leading to increased morbidity and mortality, prolonged hospital admissions, and higher healthcare costs worldwide [18]. Several mechanisms contribute to carbapenem resistance, mainly the production of carbapenemase enzymes, alteration in the outer membrane proteins or mutations in porins, and efflux pumps. Carbapenemases can hydrolyze cephalosporins, penicillins, carbapenems, and beta-lactamase inhibitors. The rapid spread of CRE is facilitated by the presence of carbapenemase genes on mobile genetic elements, like plasmids [19].

Both phenotypic and molecular-based assays for carbapenemase detection are available from cultured isolates. In clinical practice, three phenotypic method assays are currently used: (1) growth-based assays (e.g., mHT and mCIM), (2) hydrolysis methods (e.g., Carba NP and MALDI-TOF MS methods), (3) lateral flow immunoassays which detect specific antibodies of carbapenemase enzymes. Nucleic acid-based assays for carbapenemase detection directly identify the molecular determinants of carbapenemase [20]. The CPO panel, launched in 2017, can perform Gram-negative susceptibility tests and detect and classify phenotypic carbapenemases. The method involves nine test wells on a panel, each containing a beta-lactam antibiotic, alone or in combination with various beta-lactamase inhibitors, and it detects phenotypic carbapenemase and performs classification using algorithm-based detection [12]. In diagnostic laboratories dealing with numerous samples and high turnover, the ability to detect and classify carbapenemases without additional methods, in conjunction with susceptibility testing, accelerates the laboratory processes. In addition, including the carbapenemase class in susceptibility test results may help guide the antibiotic selection; for example, clinicians refrain from prescribing ceftazidime avibactam for patients infected with class B samples. Our study aims to determine the cost-effectiveness and time efficiency of a rapid diagnostic test for carbapenem-resistant bacteria in comparison to molecular-based tests. We compared the performance of the CPO panel, which we use for critical wards and ICUs in our diagnostic laboratory, with a molecular method. Our goal is to assess the effectiveness of the rapid diagnostic test and its potential to improve patient outcomes.

The accurate and rapid detection of carbapenemase is crucial for effective infection control and antibiotic management. The CPO (NMIC/ID-505) panel is used in our laboratory for Hematology, Oncology Clinics, and ICU patients and we used the Xpert Carba-R assay to assess the accuracy of the carbapenemase classification given by the CPO panel.

In recent years, there have been numerous studies in the literature about the sensitivity of the CPO panel test. However, some of these studies have reported low correct identification in Class A, some in Class B, and some in Class D. This diversity has been attributed to limited sample size, endemic carbapenemase, and strain collection diversity. Thomson et al., Park et al., Croxatto et al., Jonas et al. and Whitley et al. all have correct detection rates of 89% or higher for all three classes [12, 13, 21–23]. The first study of the performance of the CPO panel was conducted by Thomson et al. [12] in 2017. Their results showed sensitivity rates for detecting Ambler Class A, B, and D carbapenemases on 294 isolates of 97.3%, 95.6%, and 100%, respectively [12]. In a study conducted by Croxatto et al. [21], the CPO panel demonstrated sensitivity rates of 100%, 89.5%, and 95.7% for detecting Ambler Class A, B, and D carbapenemases on 185 isolates. In another study by Jonas et al. [22], the panel showed sensitivity rates of 99.5%, 97.7%, and 98.3% on 1222 isolates. Park et al. [13] reported the following rates of correct identification for carbapenemase production in 450 isolates: 98.6% for class A, 98.1% for class B, and 96.7% for class D. Whitley et al. [23] conducted a study in which they enrolled 1452 isolates of Enterobacterales, *Pseudomonas aeruginosa*, and *Acinetobacter baumannii* or *A. baumannii* complex. They found that the PPA of the CPO panel was 95.3%, 94%, and 95% for class A, B, and D, respectively. KPC-producing *K pneumoniae* is prevalent in the US, and also endemic in some parts of Europe such as Greece and Italy, and Korea [24, 25]. In the Italian-speaking region, KPC was the most commonly detected genotype (63%), in contrast to the French-speaking parts of Switzerland where OXA-48 was more common (60%) [26]. As a US company, BD Phoenix CPO panel may be more effective in detecting carbapenemases and its variants in the US.

In a study carried out in Mexico, it was found that CPO panels were 75% sensitive and 100% specific in detecting a class A carbapenemase in *K. pneumoniae* isolates (n:154). The best accuracy was observed in detecting a class A carbapenemase in *K. pneumoniae*, with an accuracy rate of 96.10% [27]. However, Ong et al. [10] found sensitivity rates to be 43.3%, 100%, and 100% for detecting Ambler Class A, B, and D carbapenemases on 190 isolates. They attributed their lower sensitivity rates for Ambler Class A to a higher rate of IMI-positive isolates than in the Thomson et al. [12] study and suggested that laboratories dealing with IMI carbapenemases should be aware of the potential limitations in detecting this specific Class A carbapenemase. Another study conducted in Belgium in 2019 on 287 isolates reported sensitivity rates of 14.3%, 82.9%, and 89.8%, respectively, for Ambler Class A, B, and D carbapenemase detection; they attributed the low susceptibility in Class A to variants in the KPC gene [28]. Simon et al. [29] reported that the CPO panel had a total Ambler test sensitivity of 79% in 95 isolates. They indicated that the highest false negative rate was observed in Class A. According to their study, the Ambler test was unreliable in detecting GES and KPC carbapenemases due to difficulties in detecting GES-type carbapenemases using colorimetric methods and KPC variances.

In our study, 16 isolates were identified as Class A by genotypic test and only 11 isolates were classified as Class A by the phenotypic test. Five Class A isolates were missed by the CPO panel. However, there was a classification error in only one of these five isolates, which was erroneously reported as Class D. Unfortunately, we couldn’t conduct sequence analysis for this isolate because of our limited budget. It is possible that the culture includes two distinct *K. pneumoniae* isolates, and different isolates may have been used for storage and subsequent molecular evaluation. The CPO panel’s algorithm was unable to classify the remaining four isolates and reported only carbapenemase producer. It is essential to note that the results of our study may have been affected by the limited number of samples. We had a restricted number of samples, and we chose CRKP isolates arbitrarily. We acknowledge that this limitation may have influenced the generalizability of our findings. PPA is calculated identically to sensitivity, and NPA is calculated identically to specificity [15]. While our PPA value was 100%, the NPA was 85.7% in Class A carbapenemases, indicating a relatively lower specificity in the CPO panel’s detection and classification. As a result, the kappa value of the CPO panel for class A was found to be in substantial agreement. It is worth noting that the presence of different KPC variants can affect the performance of the CPO panel. With more than 90 identified KPC variants, of which KPC-2 and KPC-3 are the most common clinical variants, their epidemiology varies geographically with endemic or sporadic spread [30]. Currently, there has not been a comprehensive study conducted on the various KPC variants within Turkey. In Turkey, there has been only one study conducted on the subject, which was done by Akgül et al. [31]. The study involved 216 cases of *K. pneumoniae* that were isolated from patients with positive blood cultures. The molecular analysis of the isolates showed that all of them were KPC-2-positive and belonged to ST11 variants [31]. However, due to budget constraints, we were unable to analyze the KPC variant types of our isolates. The lower sensitivity of the CPO panel in detecting and classifying Class A carbapenemases in our study might be due to local variance in Class A carbapenemases. Therefore, confirmatory tests may need to be added to the routine laboratory protocols for isolates assigned to Class A by the CPO panel. The CPO panel identifies the presence and class of carbapenemase through a phenotypic method, while the Xpert Carba-R assay detects the carbapenemase enzyme through a genotypic method. When the gene detected in the genotype is expressed, it is reflected in the phenotype. For this reason, the two test results may differ.

In a Japanese study, IMP is the primary carbapenemase type in Japan, CPO panels had a 96.7% PPA value for in 133 carbapenem-resistant and susceptible strains. All CPO panels were precisely identified as 54 IMP producers with a 100.0% PPA value [32]. During our study, we found that three isolates were classified as Class B. This classification was confirmed through both genotypic testing and the CPO panel. It’s important to note that our study had a very low sample size, which limited our findings. However, the CPO panel had a 100% PPV and a 100% NPV for Class B.

According to a study carried out in Korea, the accuracy of identifying class A, B, and D carbapenemases in 235 isolates was found to be 78.6%, 100%, and 60%, respectively. They explained the lower accuracy percentage in Class D to the small sample size of only five isolates that were tested [33]. In another Korean study of 109 clinical CPE isolates, the accuracy of identifying class A and B carbapenemases of the CPO panel isolates was 78.8% and 65.9%, respectively. In this study, 32 samples were tested for class D and the accurate identification rate was found to be 56.3%. They stated that all class D isolates were OXA-48-like enzymes and attributed the low level accuracy of class D to the difficulty in detecting class D OXA-48-like enzymes, which often causes low-level carbapenem resistance in vitro. The panel correctly classified 81.3% of *K. pneumoniae* KPC isolates to class A. It was found that the panel was unable to classify 40.0% of IMP and 63.6% of VIM isolates to class B [34]. However, Murata et al. [32] reported a PPA value of 100.0% for IMP producers. On the other hand, the Korean study by Park et al. [13] reported accurate identification of carbapenemase production in 450 isolates, with a 96.7% accuracy for class D. In our study, we identified 28 isolates that were classified as Class D by the CPO panel. Out of these, 27 were confirmed through molecular testing. One isolate was identified as Class A through genotypic analysis, but it was detected as Class D by the CPO panel. CPO panel had a 96.4% PPV and a 100% NPV for Class D.

Some studies reported the classification results of the CPO panel by including not only Enterobacterales, but also other gram-negative bacteria. Berneking et al. [35] assessed 194 isolates of CRE and non-fermentative gram-negative rods. The CPO panel correctly classified 79.17% of Enterobacterales and 67.16% of non-fermentative gram-negative rods for the classification of carbapenemases. Zhang et al. [36] conducted a study where they tested a total of 217 clinical isolates (including Enterobacterales and *A. baumannii*) out of which 178 were resistant to carbapenem while 39 were susceptible to it. They reported that the sensitivity and specificity for the CPO Ambler test were 56.71% and 94.87%, respectively. But they did not give separate results on a bacterial basis [36].

There is only one article published on this subject in our country. They reported that the CPO panel was found to be highly sensitive (98.7%) and specific (95.5%) in detecting carbapenemase production in a total of 447 Enterobacterales strains. During their research, the samples were not confirmed using the molecular method. Instead, the CPO panel was studied using a different phenotypic method, the modified carbapenem inactivation method [37]. Although the sample size was larger than our study, the presence of carbapenemase was not detected through molecular methods. Our study is the first to compare the CPO panel test with the molecular method in our country.

The first identification of OXA-48 was from a CRKP isolate in Istanbul, Turkey 2001. The local multicentre Study Group for Carbapenem Resistance (SCARE) studied bloodstream infections caused by CRKP in Turkey between 2014 and 2018. They found OXA-48 to be the most prevalent carbapenemase type (85.5%), followed by NDM (3.2%), both single carbapenemase enzymes [38]. However, in our study, despite the predominance of OXA-48 positivity in our hospital, we observed a notable rise in KPC positivity. This increase was noted under the limitation caused by a biased isolate selection. Additionally, it should be taken into consideration that our small sample size is among the limitations of our study.

To summarize, upon examining all the studies, the CPO panel faces challenges in detecting IMI and GES positivity, as well as KPC variants in Class A, IMP and VIM in Class B, and OXA-48-like enzymes in Class D. Therefore, it is advisable for countries to be aware of their endemic enzymes and variance of enzymes, and use the kit accordingly. It is worth noting that the use of CPO Panel significantly decreases the turnaround time, hands-on time, and costs, as compared to molecular methods. This can have a positive impact on antibiotic stewardship and infection control policies.

It is important to take into account several limitations when interpreting the results of this study. Firstly, the Cepheid Xpert Carba-R, like other commercial platforms, has limitations in detecting certain gene variants that may be prevalent worldwide [14]. Supplementing our results with a carbapenemase production test could be helpful. Additionally, our criteria of only utilizing isolates as meropenem resistant could have missed some CPE isolates. To conduct a more comprehensive study, other genera should be included, but in this study we only tested *K. pneumoniae* species. Lastly, the study had a small sample size and did not include any carbapenem-sensitive strains.

Currently, a comprehensive analysis of the various KPC strains in Turkey has not been carried out. Therefore, we suggest that future studies should consider the potential variation of KPC strains in Turkey. This will help in gaining a better understanding of the prevalence of KPC in Turkey and will aid in the development of effective treatment and management strategies for this concerning issue.

## Conclusion

While this phenotypic test demonstrated acceptable detection rates for Class B and D carbapenemases, a confirmatory test for Class A carbapenemases may need incorporation into the routine laboratory protocol because of its relatively low specificity.

## Data Availability

The datasets used and/or analysed during the current study are available from the corresponding author on reasonable request.

## Declarations

### Presentation

The abstract of this study was presented as a poster presentation on the 23. KLIMIK Congress held on March 13–16, 2023.

## Abbreviations

CARB: Carbapenemase producers with no specific classification
CARBA: Ambler Class A carbapenemases
CARBB: Ambler Class B carbapenemases
CARBD: Ambler Class D carbapenemases
CDC: Centers for Disease Control and Prevention
CPE: Carbapenemase-producing Enterobacterales
CPO panel: BD Phoenix CPO Detect panel
CRE: Carbapenem-resistant Enterobacterales
CRKP: Carbapenem-resistant *Klebsiella pneumonia*
D-CARB: Dual-carbapenemase producer
ESBL: Extended-spectrum beta-lactamases
EUCAST: European Committee on Antimicrobial Susceptibility Testing
ICU: Intensive Care Unit
KPC: *K. pneumoniae* carbapenemase
MALDI–TOF MS: Matrix-assisted laser desorption ionization–time of flight mass spectrometry
MDR: Multidrug-resistant
MIC: Minimum inhibitory concentration
non-CARB: Non-carbapenemase producer
NPA: Negative percent agreement
PPA: Positive percent agreement
WHO: World Health Organization

## References

[1] Muntean MM, Muntean AA, Preda M, Manolescu LSC, Dragomirescu C, Popa MI, Popa GL (2022). Phenotypic and genotypic detection methods for antimicrobial resistance in ESKAPE pathogens (review). Exp Ther Med.

[2] Papp-Wallace KM, Endimiani A, Taracila MA, Bonomo RA (2011). Carbapenems: past, present, and future. Antimicrob Agents Chemother.

[3] McKenna M (2013). Antibiotic resistance: the last resort. Nature.

[4] Bianco G, Boattini M, Iannaccone M, Bondi A, Ghibaudo D, Zanotto E, Peradotto M, Cavallo R, Costa C (2021). Carbapenemase detection testing in the era of ceftazidime/avibactam-resistant KPC-producing enterobacterales: a 2-year experience. J Glob Antimicrob Resist.

[5] Logan LK, Weinstein RA. 2017. The epidemiology of carbapenem-resistant enterobacteriaceae: the impact and evolution of a global menace. J Infect Dis. 2017;215(suppl_1):S28–S36. 10.1093/infdis/jiw282 PMC5853342 28375512

[6] Centers for Disease Control and Prevention. 2022 special report COVID-19 U.S. Impact On Antımıcrobıal Resıstance. 2022; [cited 2023 Feb 2] https://www.cdc.gov/drugresistance/pdf/covid19-impact-report-508.pdf

[7] Vrancianu CO, Dobre EG, Gheorghe I, Barbu I, Cristian RE, Chifiriuc MC (2021). Present and Future perspectives on Therapeutic options for Carbapenemase-Producing enterobacterales infections. Microorganism.

[8] Tilahun M, Kassa Y, Gedefie A, Ashagire M (2021). Emerging carbapenem-resistant Enterobacteriaceae infection, its epidemiology and Novel Treatment options: a review. Infect Drug Resist.

[9] Cui X, Zhang H, Du H (2019). Carbapenemases in Enterobacteriaceae: detection and antimicrobial therapy. Front Microbiol.

[10] Ong CH, Ratnayake L, Ang MLT, Lin RTP, Chan DSG (2018). Diagnostic accuracy of BD Phoenix CPO detect for Carbapenemase Production in 190 Enterobacteriaceae isolates. J Clin Microbiol.

[11] EUCAST. The European committee on antimicrobial susceptibility testing. Breakpoint tables for interpretation of MICs and zone diameters. Version 10.0. 2020. http://www.eucast.org

[12] Thomson G, Turner D, Brasso W, Kircher S, Guillet T, Thomson K (2017). High-stringency evaluation of the Automated BD Phoenix CPO detect and Rapidec Carba NP Tests for Detection and Classification of Carbapenemases. J Clin Microbiol.

[13] Park BY, Mourad D, Hong JS, Yoon EJ, Kim D, Lee H, Jeong SH (2019). Performance evaluation of the newly developed BD Phoenix NMIC-500 panel using clinical isolates of Gram-negative Bacilli. Ann Lab Med.

[14] Byun JH, Kim YA, Kim M, Kim B, Choi JY, Park YS, Yong D (2020). Evaluation of Xpert Carba-R Assay v.2 to detect carbapenemase genes in two hospitals in Korea. Ann Lab Med.

[15] McHugh LC, Snyder K, Yager TD. The effect of uncertainty in patient classification on diagnostic performance estimations. PLoS One; 14(5):e0217146. Erratum in: PLoS One. 2019;14(6):e0218492. 10.1371/journal.pone.0217146 PMC6530857 31116772

[16] McHugh ML (2012). Interrater reliability: the kappa statistic. Biochem Med (Zagreb).

[17] Malathi K, Anbarasu A, Ramaiah S (2019). Identification of potential inhibitors for *Klebsiella pneumoniae* carbapenemase-3: a molecular docking and dynamics study. J Biomol Struct Dyn.

[18] Prioritization of pathogens to guide. discovery, research and development of new antibiotics for drug-resistant bacterial infections, including tuberculosis [cited 2023 Feb 10]; https://apps.who.int/iris/handle/10665/311820

[19] Kundu J, Rathore S, Kanaujia R, Kansal S, Gupta A, Kaur R, Angrup A, Biswal M, Ray P. Comparative evaluation of phenotypic and genotypic methods for the rapid and cost-effective detection of carbapenemases in extensively drug resistant Klebsiella pneumoniae. Indian J Med Microbiol. 2022;S0255–0857(22)00181-5. 10.1016/j.ijmmb.2022.09.008 36229350

[20] Tamma PD, Simner PJ (2018). Phenotypic detection of carbapenemase-producing organisms from clinical isolates. J Clin Microbiol.

[21] Croxatto A, Coste AT, Pillonel T, Bertelli C, Greub G, Prod’hom G. Evaluation of the BD Phoenix™ CPO detect test for the detection of carbapenemase producers. Clin Microbiol Infect. 2020;26(5):644.e9–644.e15. 10.1016/j.cmi.2019.10.002 31634549

[22] Jonas D, Reuter S, Klassen S, Weber S, Buck M, Giani T, Rossolini GM, Grundmann H (2021). Evaluation of the BD Phoenix CPO detect panel for prediction of Ambler class carbapenemases. Sci Rep.

[23] Whitley V, Kircher S, Gill T, Hindler JA, O’Rourke S, Cooper C, Tulpule A, Denys GA (2020). Multicenter evaluation of the BD Phoenix CPO detect test for detection and classification of carbapenemase-producing organisms in Clinical isolates. J Clin Microbiol.

[24] Bonomo RA, Burd EM, Conly J, Limbago BM, Poirel L, Segre JA, Westblade LF (2018). Carbapenemase-producing organisms: A Global Scourge. Clin Infect Dis.

[25] Jeong H, Hyun J, Lee YK (2023). Epidemiological characteristics of carbapenemase-producing Enterobacteriaceae outbreaks in the Republic of Korea between 2017 and 2022. Osong Public Health Res Perspect.

[26] Ramette A, Gasser M, Nordmann P, Zbinden R, Schrenzel J, Perisa D, Kronenberg A (2021). Temporal and regional incidence of carbapenemase-producing enterobacterales, Switzerland, 2013 to 2018. Euro Surveill.

[27] Correa-León YP, Pérez-Hernández JM, Martinez-Guerra BA, Rodríguez-Noriega E, Mena-Ramírez JP, López-Gutiérrez E, López-Jácome LE, Monroy-Colin VA, Mireles-Davalos CD, Padilla-Ibarra C, Quevedo-Ramos MA, Feliciano-Guzmán JM, Pérez-Vicelis T, Velázquez-Acosta MDC, Hernández-Durán M, Garza-González E (2023). Evaluation of the BD Phoenix carbapenemase-producing organism panels for the detection of Carbapenemase Producers in Escherichia coli, Klebsiella pneumoniae and Pseudomonas aeruginosa. Diagnostics (Basel).

[28] Saad Albichr I, Anantharajah A, Dodémont M, Hallin M, Verroken A, Rodriguez-Villalobos H (2020). Evaluation of the automated BD Phoenix CPO detect test for detection and classification of carbapenemases in Gram negatives. Diagn Microbiol Infect Dis.

[29] Simon M, Gatermann S, Pfeifer Y, Reischl U, Gessner A, Jantsch J (2019). Evaluation of the automated BD Phoenix CPO detect panel in combination with the β-CARBA assay for detection and classification of carbapenemase-producing enterobacterales. J Microbiol Methods.

[30] Chen L, Ai W, Zhou Y, Wu C, Guo Y, Wu X, Wang B, Rao L, Xu Y, Zhang J, Chen L, Yu F (2022). Outbreak of IncX8 plasmid-mediated KPC-3-Producing enterobacterales infection, China. Emerg Infect Dis.

[31] Akgül Ö, Körkoca H, Bora G (2024). Analysis of antibiotic resistance of KPC-2-positive *Klebsiella pneumoniae* strains isolated from blood cultures in Van, Turkey. Eur Rev Med Pharmacol Sci.

[32] Murata M, Kosai K, Akamatsu N, Matsuyama Y, Oda M, Wakamatsu A, Izumikawa K, Mukae H, Yanagihara K (2023). Diagnostic performance of BD Phoenix CPO Detect Panels for Detection and classification of carbapenemase-producing gram-negative Bacteria. Microbiol Spectr.

[33] Cho H, Kim JO, Choi JE, Lee H, Heo W, Cha YJ, Yoo IY, Park YJ (2020). Performance evaluation of automated BD Phoenix NMIC-500 panel for carbapenemase detection in carbapenem-resistant and carbapenem-susceptible Enterobacterales. J Microbiol Methods.

[34] Lade H, Jeong S, Jeon K, Kim HS, Kim HS, Song W, Kim JS (2023). Evaluation of the BD Phoenix CPO Detect Panel for Detection and classification of Carbapenemase Producing *enterobacterales*. Antibiot (Basel).

[35] Berneking L, Both A, Berinson B, Hoffmann A, Lütgehetmann M, Aepfelbacher M, Rohde H (2021). Performance of the BD Phoenix CPO detect assay for detection and classification of carbapenemase-producing organisms. Eur J Clin Microbiol Infect Dis.

[36] Zhang A, Wang X, Liang X, Zhou C, Wang Q, Zhang J, Wang H (2021). Performance evaluation of diagnostic assays for detection and classification of carbapenemase-producing organisms. Antibiot (Basel).

[37] Ozyurt OK, Cetinkaya O, Ozhak B, Ongut G, Turhan O, Yazisiz H, Donmez L, Kuskucu MA, Midilli K, Ogunc D (2023). Evaluation of the BD Phoenix CPO detect test for the detection of carbapenemase-producing enterobacterales. Future Microbiol.

[38] Aslan AT, Kırbaş E, Sancak B, Tanrıverdi ES, Otlu B, Gürsoy NC, Yılmaz YA, Tozluyurt A, Liste Ü, Bıçakcıgil A, Hazırolan G, Dağ O, Güven GS, Akova M, Study Group for Carbapenem Resistance (SCARE) (2022). A retrospective observational cohort study of the clinical epidemiology of bloodstream infections due to carbapenem-resistant *Klebsiella pneumoniae* in an OXA-48 endemic setting. Int J Antimicrob Agents.

